# An Analysis of Food Waste Production and Behavioural Patterns Among Generation Z in Five European Countries

**DOI:** 10.3390/foods15040696

**Published:** 2026-02-13

**Authors:** Neven Voća, Francesco Donsi, Mirela Alina Sandu, Viktoria Voronova, Jana Šic Žlabur, Giovanni De Feo, Ana Virsta, Marija Klõga, Jelena Lubura Stošić, Anamarija Peter, Gina Vasile Scăețeanu, Sanja Ostojić, Ivan Brandić, Gianpiero Pataro, Dario Balaban, Darko Micić, Jona Šurić, Saša Đurović, Alessandra Procentese, Lato Pezo

**Affiliations:** 1 Faculty of Agriculture, University of Zagreb, Svetošimunska cesta 25, 10000 Zagreb, Croatia; nvoca@agr.hr (N.V.); jszlabur@agr.hr (J.Š.Ž.); apeter@agr.hr (A.P.); jsuric@agr.hr (J.Š.); 2 Department of Industrial Engineering, University of Salerno, via Giovanni Paolo II, 132, 84084 Fisciano, Italy; g.defeo@unisa.it (G.D.F.); gpataro@unisa.it (G.P.); aprocentese@unisa.it (A.P.); 3 Faculty of Land Reclamation and Environmental Engineering, University of Agronomic Sciences and Veterinary Medicine of Bucharest, 59 Marasti Blvd., District 1, 011464 Bucharest, Romania; mirela.sandu@fifim.ro (M.A.S.); ana.virsta@fifim.ro (A.V.); 4 Department of Civil Engineering and Architecture, Tallinn University of Technology, Ehitajate tee 5, 19086 Tallinn, Estonia; viktoria.voronova@taltech.ee (V.V.); marija.kloga@taltech.ee (M.K.); 5 Faculty of Technology Novi Sad, University of Novi Sad, Bul. Cara Lazara 1, 21000 Novi Sad, Serbia; jelenalubura@uns.ac.rs (J.L.S.); dario.balaban@uns.ac.rs (D.B.); 6 Faculty of Agriculture, University of Agronomic Sciences and Veterinary Medicine of Bucharest, 59 Marasti Blvd., District 1, 011464 Bucharest, Romania; ginavasile2000@yahoo.com; 7 Institute of General and Physical Chemistry, University of Belgrade, Studentski Trg 12-16, 11000 Beograd, Serbia; ostojicsanja404@gmail.com (S.O.); micic83@gmail.com (D.M.); sasatfns@uns.ac.rs (S.Đ.); 8 Institute of Biomedical Systems and Biotechnology, Peter the Great St-Petersburg Polytechnic University, Khlopina d. 11, korp. 1, Lit. A, 195251 Saint-Petersburg, Russia

**Keywords:** food waste generation, prediction, convolutional neural network, structural equation modelling, theory of planned behaviour

## Abstract

Food waste remains a global challenge, particularly among younger generations. This study examines the attitudes and behaviours of 330 Generation Z individuals (aged 18–24 years) from Italy, Estonia, Croatia, Romania, and Serbia using an extended Theory of Planned Behaviour (TPB). The TPB model was expanded to include moral social values, awareness of health risks, and good provider identity. A mixed-methods approach was applied, combining 7-day food waste diaries, visual plate-waste analysis, and self-administered questionnaires. Food recognition analysis showed that Estonian participants wasted less food per meal (3.43%) than those from Italy, Serbia, Croatia, and Romania (12.53%, 12.57%, 14.53%, and 17.18%). Nationality-specific patterns emerged: Romanians mainly discarded meat and potatoes, while participants from Estonia, Croatia, and Serbia wasted fruit and vegetables; Italians most frequently wasted fish and dairy. The extended TPB effectively predicted intentions to reduce food waste, identifying key behavioural determinants that can inform targeted interventions for young consumers.

## 1. Introduction

Food waste, along with food loss, has a significant impact on both food security and the environment on global, regional and national economies [1]. Given its impact, food waste is a major challenge for responsible business and consumer behaviours [2,3]. As a multi-layered concept, the literature offers various perspectives on the factors influencing food waste but consistently indicates that young people are more likely to engage in wasteful food consumption practises [4,5,6,7,8,9]. Generation Z, i.e., young people aged between 18 and 24 years, form a crucial group that differs from other young citizens and individuals in several ways that affect their food waste habits. These include their education, lifestyle, purchasing behaviour and the way they eat and store food, especially when they are away from home during their studies.

In almost all European countries, waste management education is integrated into the education system in some way. According to Lee et al., [10], waste education is not yet fully integrated into national curricula in all European countries. In Romania, for example, teachers often lack the necessary training and resources to teach waste management effectively. In Germany and the UK, on the other hand, waste education is firmly integrated into school and university curricula and is supported by a range of teaching materials and resources available to teachers. However, there is a growing interest among teachers in incorporating sustainability topics in their lessons. Food waste prevention has become an important issue for higher education institutions worldwide, partly due to its environmental and social impact [11,12]. In some countries, such as Estonia or Croatia, waste education is relatively new but is gaining momentum thanks to recent policy changes that prioritise circular economy principles. Waste education is mainly focussed on raising students’ awareness of the importance of recycling and reducing waste generation [13].

A number of variables are associated with young people’s food wasting habits, including age, gender, and perception, place of residence, educational background and socio-economic factors. Empirical evidence generally suggests that females are more environmentally conscious and tend to generate less waste compared to males [14,15]. However, the results on this topic are not conclusive, as some studies find no significant gender differences or even contradictory patterns [16,17,18]. Numerous studies have shown that income and age have a significant influence on food waste, with younger people generally producing more waste than older people do [19,20]. Similarly, research suggests that higher-income households are more likely to waste food than lower-income households [17,21,22], a pattern that may disproportionately affect younger members of affluent households. A study by Stancu et al. [23] claims that lower income levels are associated with greater understanding and concern about food waste. Similarly, Pandey et al. [24] reported that lower-income young people have a greater awareness of the consequences of food waste and the associated economic impact than their higher income peers do. In addition, there are significant differences in living standards and purchasing power across countries, which are influenced by factors such as the minimum wage, hourly wages for student jobs and general income levels. For example, the minimum wage and average income can vary greatly from country to country, affecting the amount and type of food waste produced. Generation Z, born between the mid-1990s and early 2010s, are characterised by their familiarity with technology as they are the first generation to have grown up with the internet and smartphones from an early age. Their unique economic behaviour and digital dexterity also influence their food consumption and waste behaviour [25].

The food waste behaviour of young people is shaped by a complex interplay of sociological, psychological, demographic and economic factors and is constantly evolving through interaction with their environment. To better understand and predict this behaviour, the Theory of Planned Behaviour (TPB) provides a well-established and empirically supported framework [26]. According to the TPB, behavioural intention is influenced by three central constructs: attitudes (personal evaluation of behaviour), subjective norms (perceived social pressure) and perceived behavioural control (PBC—belief in one’s own ability to perform the behaviour). Stronger manifestations of these components generally correspond with higher behavioural intentions and consequently with a greater likelihood of action.

Attitudes reflect the extent to which a person evaluates the behaviour positively or negatively, subjective norms refer to perceived expectations of significant others, and perceived behavioural control (PBC) refers to the perceived ease or difficulty of performing the behaviour. The Theory of Planned Behaviour (TPB) has been widely applied in food waste research, where its constructs consistently demonstrate predictive value. To increase explanatory power, researchers have extended the TPB with additional variables such as past behaviour, habits, moral norms, self-identity, knowledge, and perceived moral obligation [5,27,28,29,30,31,32]. These extensions are particularly useful in the context of food waste, where behaviour is often habitual and morally charged, and empirical studies confirm their effectiveness in analysing intentions and actions [23,33,34,35]. Frequently included variables are self-identity [36], personal norms [34,37], the “good provider” identity [36], and planning habits [23,32]. Such constructs enhance the model’s ability to capture the nuanced, context-specific nature of food waste behaviour. According to this extended model, individuals are more likely to engage in waste-reducing practices when they have favourable attitudes, perceive strong social support, and feel capable of acting. These extensions have repeatedly proven valuable in food waste research [23,33,34] and were therefore adopted in this study, as also supported by recent findings [38]. A key challenge in TPB research is linking self-reported intentions to actual food waste behaviour, which requires direct observation of consumption and leftovers. Recent developments in deep learning, particularly convolutional neural networks (CNNs), offer promising tools in this area. CNNs can identify key features in plate images before and after meals, allowing accurate food waste quantification while minimising background influence [39,40].

The aim of this study was to identify the most frequently wasted food types among young people (Generation Z) in five European countries Estonia, Italy, Croatia, Romania and Serbia and to investigate the main behavioural, attitudinal and psychosocial factors influencing their intention to reduce food waste, based on the extended Theory of Planned Behaviour (TPB), with the objective of identifying potential areas for behavioural intervention and policy development. For this reason, the study aims to use food waste diaries to analyse the variance in the quantity and category of food waste between participants and use image recognition techniques to demonstrate that the assessment of food waste from the diaries is accurate. In order to analyse the food waste behaviour of young people, it is also necessary to test the extended TPB model in the context of young people’s food waste. To do this, the influence of all TPB factors on food waste in young people aged between 18 and 24 years from all five countries analysed must be determined and to identify areas where they can make changes in their food waste behaviour and habits.

The main aim of the survey analysis was to gain deeper insights into the complexities of food waste and its relationship with participants’ behaviour. Several hypotheses were formulated accordingly: the intention to reduce food waste (I) is hypothesised to increase with stronger moral considerations for food waste reduction (MC) (H1: positive effect), a more favourable general attitude (GA) (H2: positive effect), a stronger good provider identity (GPI) (H3: positive effect, as prioritising abundance may conflict with waste reduction), stronger subjective norms (SN) (H4: positive effect), higher perceived health risks (PHR) of food consumption (H5: positive effect, as higher risks may lead to discarding food), and greater perceived personal behaviour control (PBC) (H6: positive effect).

The behaviour of reducing food waste (B) is hypothesised to be positively predicted by GPI (H7), food waste (I) (H8). In addition, GPI is hypothesised to be positively influenced by MC (H9), PBC (H10), and SN (H11).

## 2. Materials and Methods

### 2.1. Sample and Data Collection

A total of 330 participants (66 per country) were recruited across five European countries: Italy (Salerno), Estonia (Tallinn), Croatia (Zagreb), Romania (Bucharest), and Serbia (Belgrade). The sampling frame consisted primarily of young adults aged 18–24 enrolled in higher education institutions. Participants were recruited through university mailing lists, course announcements, and student networks. Consequently, the sample should be regarded as representative of a university-based subpopulation of Generation Z rather than of the general youth population in each country.

Although participants were drawn from different academic disciplines and urban settings, no stratified or probabilistic sampling procedure was applied. The sample is not intended to be nationally representative with respect to socio-economic status, educational background, or rural–urban distribution.

To reduce potential selection bias, recruitment was conducted through multiple institutional channels rather than through sustainability-focused groups or food-waste-related initiatives. Participation was voluntary and not framed explicitly around pro-environmental behaviour in recruitment materials, in order to limit the overrepresentation of individuals with particularly strong environmental attitudes. Nevertheless, self-selection bias cannot be fully excluded and should be considered when interpreting the findings.

In each of the five participating countries, data collection was coordinated by a local research team consisting of three trained researchers. These individuals were responsible for recruiting participants, providing instructions, and overseeing diary and photo documentation to ensure consistency and adherence to the protocol. Data collection took place over a six-month period, from 1 October 2022, to 31 March 2023. Each participant was instructed to keep a 7-day food waste diary and to take photographs of their plates both before and after each meal during the same period. This approach allowed for the integration of qualitative diary entries with visual documentation to precisely estimate individual food waste. Additionally, participants completed an online questionnaire designed to assess their attitudes, intentions, and self-reported behaviours related to food waste. This multi-method approach enabled a comprehensive comparison between perceived and actual food waste behaviour. Participants were given detailed instructions and examples of how to complete the food waste diary and photograph the plates. All submitted diaries and images were checked for completeness and consistency before being analysed. The use of a multi-method design (diary, pictures and questionnaire) enabled triangulation and validation of the self-reported data.

Participants were recruited through the same channels used to distribute the questionnaires—email, university mailing lists, social media and local news portals—and targeted young people aged 18–24 in each country. After expressing their interest, potential participants received a detailed information sheet and a consent form. Only those who agreed to complete the full 7-day protocol (diary, pictures and questionnaire) were included in the study. All variables included in the SEM analyses were complete; no missing data were present for questionnaire responses.

### 2.2. Food Waste Diaries

During the implementation period, participants were asked to maintain a food waste diary for duration of seven consecutive days. This diary included details of the food waste produced at each meal (it was possible to skip a day and continue the measurement the next day if there was a special occasion, such as a party, birthday, feast day, etc., as such results could distort the perception of daily food waste production in normal life. The self-reported amount of food waste was recorded in portions, and one portion was defined as one fist of an average adult person. This method is proven as a most user-friendly method of food waste on plate analysis (Supplement Figure S1). Based on data from the FAO Food Density Database [41], the average weight of a fist-sized portion of food waste was estimated to be around 250 g. Although the exact mass varies depending on the density factor of each food category, 250 g was used as a standard reference. The approximation was adopted to reduce respondent burden and facilitate consistent reporting in a diary-based setting, rather than to provide precise gram-level estimates for all food categories. This method was chosen as there is no generally accepted one-step conversion from portion size to weight in the existing literature. Participants were required to evaluate and document all food and food waste, regardless of when and how it was consumed, including meals from restaurants, home-cooked meals, fast food and snacks. Food waste was divided into 12 categories: (1) fruit, (2) vegetables, (3) processed fruit and vegetables, (4) potatoes, (5) pasta, rice, cereals, (6) meat and meat products, (7) fish, (8) milk and dairy products, (9) bread, (10) cookies, (11) prepared meals and (12) other.

The diary was completed electronically using a structured template provided in Excel format. Participants completed it daily for 7 consecutive days. Some participants were given a printable version to complete manually on request, which they later scanned and submitted their entries.

### 2.3. Food Waste Photo Images

To evaluate food waste, participants recorded images of their meals before and after eating for each meal during the same 7-day period in which they completed their diary. A Convolutional Neural Network (CNN) was used to analyse these photos. CNN is a state-of-the-art deep-learning technique that is widely recognised for its effectiveness in machine learning. It represents a special form of multilayer perceptron consisting of numerous optimised hidden layers used for classification tasks. The architecture of the CNN comprises three main layers: the convolutional layer, the pooling layer and the fully connected layer [40]. The CNN model was trained using a supervised learning approach with separate training, validation, and test datasets. Model performance was evaluated using standard classification metrics, including accuracy, precision, and recall for food category identification, demonstrating satisfactory performance across a wide range of food types. For full details on the model architecture, training procedure, dataset composition, and performance metrics, readers are referred to the original publication.

The proposed CNN model was implemented using Keras with a TensorFlow backend in Python (version 3.12). The training dataset consisted of 23,552 images organised into 157 food categories, each containing 50–200 images. Images were resized to 100 × 100 pixels to reduce computation time, and each was labelled according to its category. The dataset was randomly split into training and testing sets.

The model employed a sequential architecture with two convolutional layers for feature extraction: the first with 32 filters (3 × 3) and ReLU activation, and the second with 64 filters (3 × 3) and ReLU. Both layers used pooling with a 2 × 2 filter. The fully connected layer had 64 neurons with ReLU activation, followed by an output layer with 157 neurons using Softmax to classify images and provide category probabilities. The CNN model operates on a fine-grained classification scheme comprising 157 distinct food categories. This high-resolution categorization allows for improved recognition accuracy at the image-analysis stage. For the purposes of behavioural and statistical analysis in the present study, these 157 categories were subsequently aggregated into 12 broader food waste categories. The aggregation was performed using a predefined mapping scheme based on food type and use context (fruits, vegetables, meat, fish, bakery products, etc.), ensuring consistency between automated image recognition and the analytical framework adopted in the questionnaire and diary analyses.

The model was compiled with sparse categorical cross-entropy as the loss function and the Adam optimiser, which is efficient for noisy, large-scale optimisation problems and adaptively tunes learning rates for different hyperparameters. Training was performed for 20 epochs to prevent overfitting and optimize network parameters, including convolutional kernels and biases.

### 2.4. Food Waste Questionnaire

The questionnaire was completed immediately after the 7-day diary and image-recording phase. This order was intentional so as not to influence the behaviour of the participants during the observation period. The entire data collection period ran from 1 October 2022 to 31 March 2023. The data was collected through an online questionnaire created with Google Form software (Google Forms, Google Inc., New York, NY, USA, https://docs.google.com/). The questionnaire was developed in English and distributed to participants in Italy, Estonia, Croatia, Romania and Serbia by email via the authors’ address book, news portals and online platforms. The participants were not financially compensated. However, they received a certificate of participation and brief feedback on their personal food waste behaviour based on their submitted data, which served as an incentive.

Participants were asked to rate their level of agreement with the statements using a 5-point Likert scale, ranging from “strongly disagree” to “strongly agree” [42]. For the statements relating to objective knowledge about food waste reduction, participants were provided with three answer options: “true”, “false” and “I do not know”. A detailed table of all measurement tools can be found in the Supplement Table S1. More details regarding results of general waste knowledge and opinion data were presented in Supplement Tables S2–S4, while the results of the general attitude toward food waste were listed in Supplement Table S5. Moral criteria were extensively explored in detail due to the common feelings of guilt and negativity associated with wasteful behaviour among consumers [36,43].

To enhance respondent engagement, the questionnaire employed a varied format. It consisted of 56 questions divided into ten sections. In each section, respondents were presented with a series of 3–16 statements to which participants were asked to indicate their level of agreement. Interspersed between these agreement scales were true/false questions. This approach countered potential fatigue by offering a change in the nature of the questions and kept participants actively involved throughout the survey.

In order to minimise the influence of other questions on the answers related to food waste, the questions on food waste were asked at the beginning of the survey, which is consistent with the results of previous studies. In addition, the survey collected socio-demographic data identified in previous studies as relevant to food waste behaviour. This included information on the gender, country and living arrangement [33,34,36].

As mentioned earlier, the authors adopted TPB as a conceptual framework originally created by Ajzen [26] and developed a measurement instrument that includes four additional constructs: (1) Moral criteria; (2) Perceived health risk; (3) Financial concern; and (4) Good provider identity. Perceived behavioural control, which reflects an individual’s belief in their own ability to behave in a certain way, is another TPB construct influencing both intention and food waste behaviour. The constructs used in this study are conceptually consistent with prior research, such as Russell et al. [44] and Stancu and Lahteenmäki [45]. These studies provided guidance on the operationalisation of key constructs such as intention, behaviour, subjective norms, perceived behavioural control, and identity-related factors. Items measuring intention, behaviour, subjective norms, and perceived behavioural control were adapted and reworded from established, validated TPB-based scales in prior food waste research [44,45], while additional items, including those related to identity constructs such as the “good provider” identity, were newly developed to capture context-specific aspects of food waste behaviour. All newly created items were carefully reviewed by experts in consumer behaviour and sustainability, and the survey was pilot-tested with a small group of participants (*n* = 20) to ensure clarity, relevance, and comprehensibility. Participants were asked to complete the survey and provide feedback on clarity, comprehension, and response burden. Based on pilot feedback, several minor wording adjustments were made to improve item clarity, and the layout of the questionnaire was modified to reduce potential response fatigue. Wording changes were minor and aimed at ensuring consistency with local dietary habits, terminology, and social norms, while preserving the theoretical meaning of the constructs. For example, phrases referring to “family meals” or “household food provision” were adapted to match common usage in each country. No changes were made to the underlying constructs or scale structure.

In this study, perceived behavioural control refers to the extent to which participants believe they have control over reducing food waste. Subjective norm, a construct of TPB, refers to the social pressure individuals feel to engage in or avoid specific food waste behaviour. Financial concerns regarding the cost of food purchased but wasted can also significantly influence food waste behaviour. On the other hand, perceived health risks associated with consuming questionable food can negatively influence food waste behaviour. The concept of “good provider identity” is a sociological phenomenon that often exerts a negative influence on food waste prevention, as some consumer’s associate food with providing for other household members.

Participants submitted their completed diaries and pictures of meals (photos of plates before and after eating) electronically. The files were uploaded via a secure cloud-based folder (e.g., Google Drive, WeTransfer) or sent directly by email to the local research coordinators.

### 2.5. Statistical Analysis

The statistical software IBM SPSS (version 21) was used to analyse the questionnaire results. Cronbach’s alpha coefficient was used to measure the internal reliability of the scales for assessing psychological constructs [46,47]. If the internal reliability was above 0.7, the mean was calculated and used for further analyses.

To assess cross-country differences in food waste quantities, a one-way ANOVA was conducted followed by post hoc pairwise comparisons using Tukey’s HSD. This procedure allowed us to identify which countries differed significantly in total food waste percentages and by waste type. Statistical significance was set at *p* < 0.05.

Univariate data analyses and regression analyses were also performed. Prior to data analysis, the reliability of the measurement scale was assessed using the Cronbach’s alpha coefficient, while structural equation modelling (SEM) was used to test the model using the IBM Amos statistical programme. The structural modelling method was used to test the hypothetical model to be analysed. This method is a further development of multiple regression analysis, which can provide a more meaningful and valid interpretation of the results than alternative methods. In contrast to multiple regression, SEM allows the metric properties of the individual variables and their relationships to be analysed simultaneously, which makes it much easier to interpret the results. SEM can analyse more complex models, such as the extended TPB model used in the present study.

## 3. Results

### 3.1. Socio Demographic Results

The study involved 330 respondents from Generation Z (aged 18–24), with 66 participants from each of the five countries: Estonia, Italy, Croatia, Romania, and Serbia. The general demographic parameters of the survey participants are listed in Table 1. According to the data collected, a total of 39.2% male participants and 60.8% female participants were included in the survey. The majority of participants lived with their family (44.9%) or with roommates (other participants) (37.4%).

### 3.2. Results of the Participants’ Diary and Food Waste Quantity Evaluated Using the CNN Model

Several studies have explored the use of diary methods to assess food waste at the household level, highlighting both their potential and limitations. Giordano et al. [48] conducted a pilot study in Italian households to evaluate the reliability of questionnaires as a tool for measuring food waste, emphasising the challenges of self-reported data. Similarly, Quested et al. [49] compared diary-based food waste records with waste compositional analysis, providing valuable insights into the accuracy and practicality of diary approaches in capturing household food waste. In this study, the average quantity of food waste during the seven-day period (measured by the participant’s fist) as recorded in the participant’s diary is presented in Table 2. Within this investigation, the goal was to estimate and contextualise food waste generation across five European countries, rather than focusing on socio-demographic characteristics.

Although intentions and Good Provider Identity (GPI) were significant predictors of self-reported food waste behaviour, correlations with objectively measured waste (via diary and CNN analysis) were moderate (r = 0.457–0.496). This discrepancy reflects the well-documented attitude–behaviours gap in environmental behaviours research. Strong intentions do not always translate into proportional reductions in actual waste due to multiple factors: habitual patterns of food use, situational constraints such as unexpected guests or over-purchasing, and household characteristics that modulate control over waste. Measurement differences may also contribute to the gap; self-reported behaviours can be influenced by social desirability and recall bias, whereas diary/CNN methods provide more objective but possibly context-limited snapshots of waste. Taken together, these results highlight that intentions alone are insufficient to fully predict food waste behaviours. Interventions should therefore not only target motivational constructs but also address structural and behavioural barriers that limit the enactment of waste-reduction intentions.

Table 3 presents the differences in food waste estimates obtained from participant diaries and image collections across the surveyed countries. It also shows the correlation coefficients between the two methods, providing insight into the consistency and agreement between self-reported and image-based food waste assessments.

### 3.3. Participants’ Food Waste Survey Results

Food waste behaviour and consumer motivations are shaped by complex sociological, psychological, demographic, and economic factors that evolve through continuous interaction with the environment, which was a motive to perform the study of food waste behaviour for Generation Z in five European countries (similar to Attiq et al. [50] and Novitasari et al. [51]). The findings reveal notable cross-country differences in knowledge, attitudes, and behaviours, suggesting opportunities for targeted interventions and policy measures. Table 4 presents the descriptive analysis and ANOVA table of constructs derived from the questionnaire on food waste behaviour. The food waste behaviour is characterised by several constructs towards food waste including general waste knowledge (GWK), general attitude (GA), moral criteria (MC), intention (I), behaviour (B), perceived behaviour control (PBC), subjective norm (SN), financial concern (FC), perceived health risk (PHR), planning habit (PH) and good provider identity (GPI). For each category and country, Table 4 provides the average score for each construct. In addition, the behaviour and motivations of everyday food consumers are influenced by a complex set of sociological, psychological, demographic, and economic factors. This complex behaviour is subject to constant change due to constant interaction with the environment.

Cross-country differences can be partially explained by contextual factors [52,53,54,55,56,57,58,59]. Estonia’s low food waste aligns with national circular economy policies, strong household recycling infrastructure, and widespread awareness campaigns. In contrast, higher waste rates in Romania may reflect less developed waste management systems, different dietary patterns, and economic factors such as lower disposable income and higher relative meat consumption. Italy’s higher fish waste corresponds to local culinary preferences and the frequent purchase of perishable seafood items. These contextual explanations complement the statistical findings and help interpret the observed cross-country variation.

### 3.4. SEM Assumptions

Structural equation modelling (SEM) was used to assess the consistency between the expressions used in the study and the constructs. The SEM analysis comprises two stages. In the first stage, the measurement model is assessed, which clarifies how each construct is measured with the corresponding expressions. In this stage, the observed variables of the constructs are analysed together and loading values, construct reliability, internal consistency and discriminant validity are determined. The 56 items (study questions) were loaded as individual factors.

The study tested four important assumptions for the SEM, including multivariate normality, multicollinearity, sample size and positive definiteness. Multivariate normality was assessed by regression analysis, which revealed that 0 out of 330 participants were outliers according to Mahalanobis distances [60]. The multicollinearity assumption was not violated [61], with variance inflation factors (VIF) less than 10 (1.700–8.643) and tolerances greater than 0.01 (0.064–0.588). The linearity assumption was not assessed due to the use of a Likert scale. Normality assumptions were not violated, with Kolmogorov–Smirnov test statistics greater than 0.05 (0.179–0.493) for all variables [62]. In addition, the assumptions of linearity and homoscedasticity were tested and found not to be violated. The variance values were checked and all variables had similar variance levels (0.223–1.681).

The sample size was also checked using an online calculator (Free Statistics Calculators, version 4.0). The analysis showed that a minimum sample size of 190 participants was required to achieve a desired statistical power of 0.8, taking into account 10 latent variables and 56 observed variables, with an expected effect size of 0.3 and a probability level of 0.05. In addition, a minimum sample size of 148 participants was required for the model structure.

#### 3.4.1. Exploratory Factor Analysis

To determine the number of constructs that could be derived from the survey data, an exploratory factor analysis (EFA) was conducted using the principal component method [63]. To ensure an accurate interpretation of the factors, the factors were rotated using the varimax procedure. Kaiser–Meyer–Olkin (KMO) [64] and Bartlett’s test of sphericity [65] were performed during the EFA. The resulting factors were evaluated using agreement indicators, and any coefficients below 0.3 were suppressed in Supplement Table S6. The assumption of positive definiteness was not violated, as the determinant value was greater than zero (3.570 × 10^−22^). The KMO value was calculated as 0.930, which indicates a strong interdependence of all variables. The sphericity was not significant (*p* < 0.001), so that a factor analysis could be performed. The EFA results showed that nine eigenvalues were greater than 1, indicating that the SEM model would have ten constructs. According to the EFA, ten variables (i.e., nine constructs) were found to be suitable for the SEM model towards Food waste due to the high factor scores: GA, B, FC, GPI, I, MC, PBC, PHR, SN.

#### 3.4.2. Individual Construct Reliability

The individual construct reliability testing [66] was demonstrated on individual constructs in accordance with the Cronbach’s alpha coefficient (Table 5).

The factor loadings exceed 0.6, and according to Table 5, Cronbach’s alpha values [67] surpass 0.70. All these values are more significant than the recommended reliability thresholds, suggesting that all proposed constructs were reliable to represent variables and the data within the SEM model [68].

The Cronbach’s alpha for GWK is relatively low (α < 0.7), indicating poor internal consistency among the 19 items in this construct. However, the construct reliability (CR) value is high (0.952), suggesting that despite the low alpha, the items collectively measure the intended construct reliably. The PH construct shows acceptable internal consistency and reliability, although the AVE (0.612) indicates modest convergent validity. These constructs were excluded from further TPB calculations. According to Table 5, the GA construct shows good internal consistency (α > 0.7), with a moderate average variance extracted (AVE) (0.590), indicating that the items share a reasonable amount of the variance within the construct. The MC construct demonstrates excellent internal consistency and reliability, with a high AVE (0.771) suggesting strong convergent validity. The I construct exhibits excellent internal consistency and reliability with a relatively high AVE (0.727), which indicates good convergence of the individual items. The B construct shows strong internal consistency and reliability with a moderate AVE (0.678), which indicates good convergent validity. The PBC construct shows excellent internal consistency and reliability, with a relatively high AVE (0.734), indicating strong convergent validity. The SN construct shows excellent internal consistency and reliability with a high AVE (0.775), indicating robust convergent validity. The FC construct shows good internal consistency and reliability with a relatively high AVE (0.736), indicating adequate convergent validity. The PHR construct shows good internal consistency and reliability with a moderate AVE (0.683), indicating adequate convergent validity. The GPI construct shows excellent internal consistency and reliability with a high AVE (0.810), indicating strong convergent validity.

Although some constructs show lower internal consistency (GWK and PH), most constructs show overall good to excellent reliability and validity based on Cronbach’s alpha, AVE and CR. Constructs with lower AVE values could benefit from further examination of their item composition in order to improve their measurement properties.

#### 3.4.3. Confirmatory Factor Analysis

To evaluate the unidimensionality of the variables, a confirmatory factor analysis (CFA) [69] was performed for all survey variables. Each statement was treated as a separate variable, with the participants’ responses serving as input variables. The underlying assumption in conducting the factor analysis is that the data are interdependent. As recommended by Coakes [70] and Siyambalapitiya et al., [71], a variable is considered to belong to a specific factor if its loading on that factor exceeds 0.3. The reliability of the individual constructs and the convergent and discriminant validity of the measurement model were assessed using the CFA. The standardised regression weights for each variable forming the specific construct are shown in Supplement Table S7. To establish convergence validity, the average variance extracted (AVE) should exceed 0.5 and the construct reliability (CR) should be above 0.7, as suggested by Fornell and Larcker [72]. Table 5 contains the AVE and CR calculations for the constructs included in the structural equation modelling (SEM) model.

#### 3.4.4. Convergent Validity

Convergent validity is an additional test of construct validity, as it studies if the correspondence or convergence between the constructs exists [73]. Construct validity necessitates both discriminant and convergent validity (Supplement Table S8).

Discriminant validity, Supplement Table S8 requires the AVE to be at least 0.50, and the square roots of the AVE values should exceed the correlation values between the latent constructs. For convergent validity, the CR values of the entire scale should be extensive [74]. In PLS-based structural equation modelling, a CR value above 0.70 is expected for scale reliability [75]. According to Supplement Table S8, the discriminant validity was reached for all constructs.

#### 3.4.5. Correlation Analysis Between Constructs

The assumed paths, connecting constructs intention, food waste behaviour and good provider identity were marked by bounding lines in Supplement Table S9.

#### 3.4.6. SEM Model

CFA is used to compute the fit indices in SEM model. These indices then present the overall model fit to the data. If the model is deemed acceptable, specific paths should be examined to determine whether they are statistically significant or not. The FC construct was excluded from the SEM analysis according to insignificant *p*- values.

The SEM model was used to test the set of hypotheses, proposed earlier in the text, (Supplement Table S10). This calculation allows for the examination of the overall fit of the model to the data and the simultaneous computation of all path coefficients [74,76].

**Figure 1:**
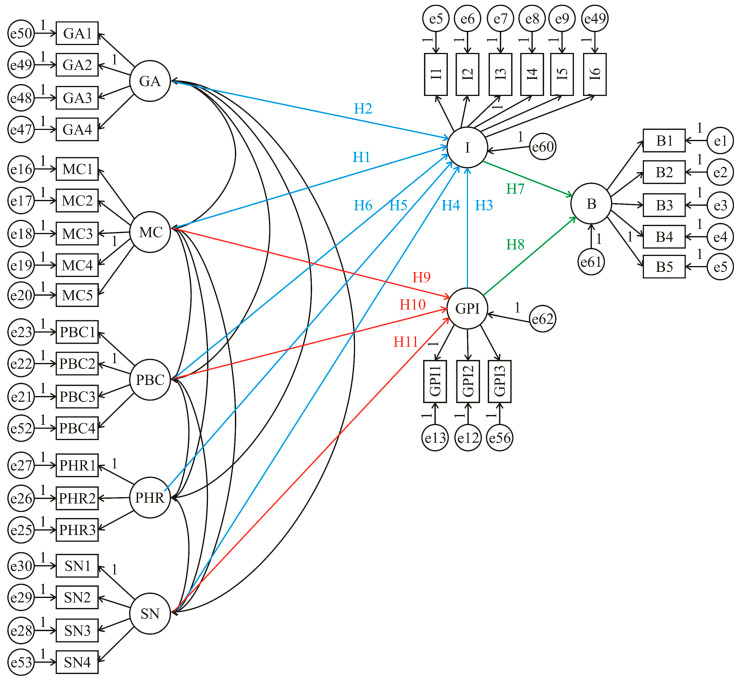
Correspondence analysis presentation of average 7-day food waste measured by participant’s diary/image collection, expressed by portion.

The proposed model and hypotheses were evaluated using structural equation modelling, Figure 1.

It is obvious that the reduction in food waste is a complex behaviour that is influenced by several factors. This was confirmed by the SEM analysis, which proves that different factors can predict the intention to reduce food waste (I). These factors include: MC (β = 0.458; S.E. = 0.079; *p* < 0.001), GA (β = 0.098; S.E. = 0.043; *p* = 0.019), GPI (β = 0.357; S.E. = 0.070; *p* < 0.001), SN (β = 0.247; S.E. = 0.068; *p* < 0.001), PHR (β = 0.733; S.E. = 0.088; *p* < 0.001) and PBC (β= −0.209; S.E. = 0.081; *p* = 0.010), all of which were confirmed by SEM analysis (defined as hypotheses H1, H2, H3, H4, H5, and H6, respectively). Furthermore, the analysis suggests that the food waste behaviour of reducing food waste is influenced by both GPI (β = 0.175; S.E.= 0.086; *p* = 0.042) and I (β = 0.146; S.E. = 0.062; *p* = 0.018), as confirmed by SEM analysis (defined as hypotheses H7 and H8, respectively). Additionally, GPI appears to be influenced by MC (β = 0.273; S.E. = 0.064; *p* < 0.001), PBC (β = 0.640; S.E. = 0.067; *p* < 0.001), and SN (β = 0.118; S.E. = 0.059; *p* = 0.047), as supported by SEM analysis (defined as hypotheses H9, H10, and H11, respectively).

Contrary to typical TPB expectations, perceived behavioural control (PBC) exhibited a negative effect on intention to reduce food waste (β = −0.209). One possible explanation is that participants with higher PBC may perceive themselves as already capable of managing their food effectively, thereby reducing the perceived need to consciously form strong intentions to minimise waste. In other words, high PBC could reflect a sense of self-efficacy that paradoxically decreases the motivational urgency to plan or commit to waste-reduction behaviours.

Another potential interpretation is that the PBC scale may partly capture complacency or overconfidence regarding food management, which can weaken the intention formation process. These findings are consistent with prior research suggesting that habitual behaviours, cultural norms, and identity-related factors can modulate or even reverse expected TPB relationships [77,78]. Future research should consider including measures of habitual behaviour or perceived urgency to better disentangle these effects.

Supplement Table S10 confirms the relationship between the constructs in the SEM model, based on the low *p*-values (*p* < 0.001 and *p* < 0.05, indicating a statistically significant relationship). The C.R. values for most relationship are well above 2, further supporting the strength and reliability of these relationships. To summarise, the SEM model confirms multiple strong relations between different constructs (I, MC, GA, GPI, SN, PHR, PBC and B) multiple times in the study, with strong statistical significance observed in most cases. The confirmatory factor analysis (CFA) yielded favourable fit index values for the measurement model (χ^2^/df: 2.394, GFI: 0.898, AGFI: 0.850, NFI: 0.916, TLI: 0.913, CFI of 0.910, and RMSEA of 0.037 [79].

#### 3.4.7. Mediator Effects

According to the SEM analysis, the investigation for mediating effect of the constructs can be performed when path “a” and path “b” both demonstrate statistical significance.

The Sobel method and its variations adopt an approach where a statistical test is conducted on the product of variables “*a*” and “*b*”. According to Sobel [80] and Gu et al. [81], standard error can be estimated using the following equation, utilising unstandardized estimates for “*a*” and “*b*”:

(1) $$SE_{ab}=\sqrt{a^{2}\cdot SE_{b}+b^{2}\cdot SE_{a}}$$

Once the standard error estimate is obtained, a z-test can be performed by dividing the value of *a*·*b* by the standard error:

(2) $$z=\frac{a\cdot b}{SE_{ab}}$$

In case of *p* < 0.05, the critical z-value is conventionally 1.96; and any z-value exceeding this threshold indicates support for the mediation hypothesis. According to performed calculation (Supplement Table S11), GPI was mediator between PBC and B, while I was acting as a mediator between MC and B, PHR and B, and between GPI and B.

The mediation analysis was supplemented with a nonparametric bootstrapping procedure using 5000 resamples [82]. Bias-corrected 95% confidence intervals (CIs) were estimated for all indirect effects. The results of the bootstrapping analysis were consistent with those obtained using the Sobel test, thereby confirming the robustness of the identified mediation pathways.

Specifically, the indirect effect of moral considerations (MC) on food waste reduction behaviour via intention was statistically significant, with a bootstrapped indirect effect of β = 0.067 and a 95% CI [0.032, 0.109], which did not include zero. Similarly, the indirect effect of good provider identity (GPI) on behaviour through intention was positive and significant (β = 0.052, 95% CI [0.018, 0.091]). The mediation pathway from subjective norms (SN) to behaviour via intention was also supported, with a bootstrapped indirect effect of β = 0.036 and a 95% CI [0.011, 0.068].

In contrast, the indirect effect involving perceived behavioural control (PBC) and intention was negative but statistically significant (β = −0.030, 95% CI [−0.062, −0.007]), further corroborating the unexpected direction of the PBC–intention relationship observed in the SEM results.

## 4. Discussion

### 4.1. Food Waste Evaluation According to Dairy and Image Data

Table 2 presents the average 7-day food waste recorded by participants using diary entries, expressed in portions of food waste (FW), alongside the food waste quantity estimated using the CNN model based on participant image collections over the same period, expressed as a percentage of FW. These results reveal that food waste generation varies among the young people from the different nations studied.

The highest amount of food waste was reported by participants from Romania (5.39 portions per 7-day period), Croatia (5.26 portions per 7-day period) and Serbia (5.16 portions per 7-day period), although there was no significant difference between the amount of food waste generated in these countries. The significantly lower amount of food waste was recorded by the Italians with 3.66 portions per 7-day period, while the participants from Estonia had the lowest amount with only 1.40 portions per 7-day period. In terms of food waste, meat and meat products are thrown away the most in Romania and Estonia (1.06 and 0.23 portions per 7-day period), while in Italy fish waste (1.28 portions per 7-day period) is thrown away the most. On average, participants in Serbia threw away large amounts of vegetables (0.93 portions per 7-day period), while participants in Croatia discarded relevant amounts of fruit (1.25 portions per 7-day period).

The meat and meat products category accounts for the largest proportion of food waste during the survey. It has an average food waste share of 0.59 food waste portion and 1.72% of total food waste on the plate. This shows that meat and meat products contribute significantly to the overall proportion of food waste. Prepared meals follow closely behind with an average of 0.44 of total food waste and 1.58% of food waste on the plate. This suggests that prepared meals also contribute significantly to food waste, possibly due to their perishability and the tendency to throw away leftovers. Fruit and vegetables also contribute significantly to food waste with an average share of 0.70 and 0.51 respectively. They account for 1.53% and 1.47% of food waste on the plate. Processed fruit and vegetables have an average portion of 1.18, but their share of total food waste is relatively low at 0.45%. This indicates that although processed products are thrown away in considerable quantities, they do not have a significant impact on the overall proportion of food waste. Fish also makes up a considerable proportion of total food waste with an average share of 0.33 and a share of 1.18%. Potatoes account for 0.94 of total food waste with an average portion of 0.31, indicating that starchy vegetables are another common category of discarded food. Pasta, rice and cereal products have an average share of 0.29 and contribute to 0.87% of total food waste, showing that staple foods are also frequently thrown away. Milk and dairy products, bread, biscuits and other fractions have lower average shares and percentages of total food waste [83]. To summarise, meat and meat products, ready meals, fruit and vegetables account for the largest proportion of food waste, both in terms of portion sizes and as a percentage of total food waste on the plate [84].

The graphical representation of correspondence analysis for the experimental results is illustrated in Supplement Figure S2. Significant correspondence was ascertained amid the explored participants’ food waste habits (from Italy, Estonia, Croatia, Romania, and Serbia), (total inertia was 0.366; χ2 was 7.621; df = 44; *p* < 0.00004). The obtained eigenvalues were 0.263 and 0.076, with the first two dimensions explaining 90.16% of the total inertia, effectively utilising a substantially substantial portion of the raw information.

Supplement Figure S2 shows that the Romanian participants predominantly generated food waste from meat and meat products, potatoes, cookies, and prepared meals, while the participants from Croatia and Serbia tended to generate food waste from fruit, vegetables, processed fruits and vegetables, pasta, rice and cereals [85]. Conversely, the Italian participants primarily produced food waste in the form of fish or leftover dairy products such as milk and cheese [86]. The participants from Estonia mainly left predominantly fruits and vegetables [87].

The comparison between the results obtained from the participants’ diaries and the analysis of the participants image collections reflects the division into two pathways in food waste calculation: (1) human estimation and (2) artificial neural network (ANN) modelling. Both approaches have shortcomings; human estimation can be subjective, while CNN modelling has limitations in predicting food waste [40].

The results obtained can be partly explained by the potentially excessive eating habits of the individual nationalities involved in the study. It is evident that Romanian participants generated the largest amount of food waste from the category of meat and meat products, suggesting that this type of food may be more frequently present in their daily diet.

The results of the percentage of food waste remaining on the plates, measured by the participants’ image collection, are also presented in Table 2. When calculating the average food waste among the participants, it was found that 12.05% of the food was wasted, indicating a relatively high proportion. However, a more comprehensive analysis should be conducted to determine whether the images captured both edible and non-edible parts of the food. It is evident from Table 2 that the model shows slight deviations from the participants’ food waste diaries. The highest percentages of total food waste were recorded among participants from Romania (17.18%) and Croatia (14.53%), followed by those from Serbia (12.57%) and Italy (12.53%). In contrast, the data indicate that young people from Estonia left significantly lower amounts of food waste on their plates (3.43%) compared to their peers in Croatia, Romania, Italy, and Serbia.

These patterns can be partially explained by contextual factors, such as dietary preferences, local food prices, and household purchasing practices. Estonia’s low waste aligns with its strong circular economy policies, widespread recycling infrastructure, and public awareness initiatives [87]. In contrast, Romania’s higher waste may reflect limited waste infrastructure and lower household-level awareness. Cultural dietary habits also shape waste composition: Italian households purchase fresh seafood more frequently, which is highly perishable, while Romanian households consume more meat, leading to higher waste of this category. Additionally, economic factors such as food prices and disposable income may modulate purchasing and waste behaviours [88].

### 4.2. Country-Level Differences in Food Waste: Diary vs. Image-Based Analysis

The comparison between the estimates of food waste obtained from the participants’ diaries and the analysis of the participants’ image collections analysis was analysed by correlation (Table 3). All correlations were statistically significant, at a *p* < 0.05 level. The highest correlation between the participants’ diaries and the analysis of the participants’ image collections was found for fish waste (0.999), while the lowest correlation was found for the food waste category pasta, rice, cereals (0.708).

The ranking of the differences between various countries participants’ diaries and the analysis of the participants’ image collections for different food categories were also evaluated. The participants from Estonia gained the best scores in almost all food categories: for fruits, vegetables, potatoes, pasta, rice, and cereals, meat and meat products, fish, milk and dairy products, bread, cookies and prepared meals, other (best) and overall results (worst), for food waste percentages in the diaries and for pictures. According to diary data, Estonia was again the best score for processed food and vegetables food waste reduction, while the image collection of Estonian participants obtained the best score in other food waste category [52,53]. Although the results in the diaries and pictures were worst for Romania the overall correlation between diaries and pictures was 0.8 each.

All correlations were statistically significant, at a level of *p* < 0.05. The highest correlation between the participants’ diaries and the analysis of the image collections was obtained for fish waste (r = 0.999), while the lowest correlation was obtained for the category of food waste from pasta, rice and cereals (r = 0.708). According to these data, the amount of food waste documented in the diaries correlates well with the data collected using image recognition techniques.

### 4.3. Food Waste Survey Results

The food waste behaviour and motivations of everyday food consumers are influenced by a range of complex sociological, psychological, demographic, and economic factors [52,53,54,55,56,57]. This complex behaviour is subject to constant change through ongoing interaction with the environment. The findings suggest notable differences in waste-related knowledge, attitudes, food waste behaviour, and other factors across the countries studied, highlighting potential areas for targeted intervention or policy action [52,53,54,55]. These variations may reflect differences in cultural norms, levels of environmental awareness, accessibility of food waste infrastructure, or national policies and educational campaigns. The countries with established awareness programmes and food waste regulations may demonstrate more responsible food handling and lower levels of waste. Understanding the social and cultural context of each country is therefore essential for designing effective strategies to influence behaviour and reduce household food waste. This also emphasises the importance of integrating both individual-level and systemic approaches to food waste prevention.

Most observed constructs in the SEM model demonstrated good to excellent reliability and validity. Constructs such as MC, I, B, PBC, SN, FC, PHR, and GPI also showed high AVE values, indicating strong convergent validity [89]. Confirmatory factor analysis confirmed unidimensionality and supported the reliability and validity of the measurement model, as most AVE and CR values met recommended thresholds [90]. Discriminant validity was also established. SEM model (Figure 1) was used to visualise the hypothesised paths, confirming model fit and significance of selected relationships.

The SEM analysis confirms that food waste reduction is a complex behaviour influenced by multiple psychological and contextual factors. Several constructs significantly predict the intention to reduce food waste, including moral considerations, general attitudes, social norms, perceived health risks, and perceived behavioural control [91]. Both intention and identity as a good provider play a role in shaping actual food waste behaviour [23]. The good provider identity itself is further shaped by factors such as moral considerations, social norms, and perceived behavioural control [92]. These relationships are statistically significant and supported by confirmatory factor analysis, which indicated a good overall model fit. Overall, the SEM model validates the strong interconnections among these constructs in explaining food-waste-related behaviours.

The integrated multi-method approach employed-comprising food waste diaries, image recognition techniques, and data from a self-administered survey enabled a comprehensive examination of attitudes, moral values, perceived behavioural control, and subjective norms, thereby providing a foundation for the development of targeted interventions. This understanding facilitates the design of tailored educational programmes aimed at increasing awareness of food waste and promoting more responsible consumption behaviours among young individuals. These findings offer valuable insights for the formulation of strategies intended to strengthen practical competencies such as meal planning and portion control. The determinants identified through the application of the Theory of Planned Behaviour (TPB) may be utilised in initiatives focused on enhancing food storage practices. These educational and skill-building strategies are designed to address food waste at the individual level, while also contributing to the achievement of broader sustainability objectives.

### 4.4. Country-Level Differences in Food Waste: Diary vs. Survey Based Data

The correlation analysis between TPB constructs and food waste data, derived from both diary-based self-reporting and CNN-based image analysis across five countries (Italy, Estonia, Croatia, Romania, and Serbia), reveals consistently strong and positive associations (Table 6). All correlations were statistically significant, at a level of *p* < 0.05.

The TPB construct Intention (I) shows the highest correlations with food waste: for diary data, r = 0.754 (Italy), 0.768 (Estonia), 0.753 (Croatia), 0.769 (Romania), and 0.762 (Serbia); for CNN image analysis, r = 0.754 (Italy), 0.761 (Estonia), 0.753 (Croatia), 0.767 (Romania), and 0.769 (Serbia). Similarly, MCs are strongly correlated, ranging from r = 0.659–0.681 (in diaries study) and 0.641–0.676 (in CNN data). PBC shows robust associations to diary study, with r = 0.666 (Croatia) to 0.690 (Italy), and with CNN data, from r = 0.671 (Croatia) to r = 0.704 (Romania). PHRs are also strongly linked to diary data, with r = 0.680 (Croatia) to 0.697 (Estonia), while the correlation coefficient to CNN data reaches r = 0.676 (Italy) to r = 0.693 (Estonia). GPI constructs display similar relation to diary data, r = 0.681 (Croatia, Serbia) to r = 0.695 (Romania), whilst relation to CNN data reaches r = 0.677 (Estonia) to r = 0.708 (Italy). Other constructs, like SN, shows slightly weaker but still positive correlations, with SN ranging from 0.656 to 0.678 (diary) and 0.648–0.667 (CNN), and FC from 0.533 to 0.568 (diary) and 0.547–0.569 (CNN).

In contrast, weaker positive correlations for the correlation between B and objectively measured food waste (via diaries and CNN image analysis) was measured, suggest that actual self-reported behaviours (e.g., shopping planning, leftover use) are less strongly aligned with the measured waste, possibly due to social desirability bias or incomplete behavioural capture. For diary data, the highest correlation is observed in Croatia (r = 0.496), followed by Serbia (r = 0.486), Italy (r = 0.482), Romania (r = 0.471), and Estonia (r = 0.471). For CNN image analysis, the highest values of correlation coefficient are again in Serbia (r = 0.482) and Romania (r = 0.470), while Italy follows closely (r = 0.469), then Estonia (r = 0.465), and finally Croatia (r = 0.457).

The observed negative relationship between perceived behavioural control (PBC) and intention appears to contradict typical TPB assumptions, which usually predict a positive association. This unexpected direction may be context specific and could reflect unique characteristics of the sample or behaviour under study. For example, participants with higher PBC may perceive that reducing food waste is easy and manageable and thus may feel less urgency or motivation to explicitly form strong intentions, whereas those with lower PBC may consciously intend to reduce waste but feel less capable of doing so. The measurement or phrasing of PBC items could contribute to this effect if items inadvertently capture a sense of complacency rather than control.

Notably, all TPB constructs are positively correlated with both forms of waste measurement, indicating that individuals expressing higher pro-environmental attitudes and intentions also tend to be associated with higher recorded food waste. This counterintuitive trend suggests a potential attitude–behaviour gap or heightened self-awareness in reporting among environmentally conscious individuals, emphasising the need for more nuanced interventions that go beyond intention and knowledge to promote actual behavioural change.

### 4.5. Implications, Limitations of the Study and Future Research

The integration of TPB-based survey data, food waste diaries, and CNN image analysis offers several important implications for both research and practice. The triangulation of these methods strengthens the reliability of food waste assessments by combining subjective self-reports with measures that are more objective. The high correlations between TPB constructs—particularly intention, perceived behavioural control, and subjective norms—and both diary and CNN data (r > 0.75 for intention in Romania and Serbia across methods) suggest that food waste behavioural intentions are strong predictors of actual food waste behaviour across multiple national contexts. These findings provide valuable insight for policymakers, indicating that strategies targeting attitudes, control perceptions, and social norms can effectively reduce household food waste [23].

However, the study is not without limitations. The reliance on self-reported data in both surveys and diaries may still be prone to social desirability bias, despite cross-validation with CNN analysis. Additionally, the image-based CNN analysis, while innovative, may not capture all nuances of waste quantity, food type, or context of disposal (composted vs. trashed). Another limitation lies in the sample size and representativeness across the five countries, which may affect the generalizability of findings. Cultural factors and differing levels of familiarity with digital tools may also influence diary accuracy and image data collection.

In comparison with the study by Graham-Rowe et al. [36], who showed that an extended TPB model predicts household food waste intentions but explains little variance in behaviour, this study focuses on Generation Z across five European countries and identifies nationality-specific patterns in both the amount and type of food wasted. The TPB framework was expanded by including moral values, health-risk awareness, and good provider identity, which significantly predict both intention and behaviour among young consumers. Using a mixed-methods approach combining questionnaires, 7-day diaries, and visual plate-waste analysis, the richer behavioural insight than survey-based studies alone was presented.

Although TPB constructs generally predicted intentions, Subjective Norms exhibited a weaker effect than expected. This may reflect cross-cultural differences in the influence of social norms on Generation Z. In more individualistic countries, young adults may prioritise personal attitudes, perceived behavioural control, or identity-based motivations over social expectations, whereas in more collectivist settings, normative pressures may play a larger role. Moreover, the inclusion of identity-related constructs, such as Good Provider Identity, and moral values may partially mediate or substitute for the influence of SN, suggesting that traditional TPB components alone do not fully capture the motivational complexity of food waste behaviour.

This study has several limitations that should be considered when interpreting the results. First, the cross-sectional design limits the ability to draw causal inferences between psychological factors and food waste behaviour. The relatively small group of respondents and their uneven distribution across countries may reduce statistical power and affect the generalisability and comparability of the findings. The use of self-reported data may be influenced by social desirability bias, potentially leading participants to underreport food waste or overstate sustainable behaviours. Despite careful rephrasing, some survey items may not perfectly capture the theoretical constructs they intend to measure, which could affect construct validity, particularly for perceived behavioural control and subjective norms. Certain relationships observed, such as the negative association between perceived behavioural control and intention may be context specific. Cultural and socio-economic factors that could influence food waste behaviours were not fully controlled or explored, and the study focused primarily on young consumers, limiting the applicability of the results to other demographic groups. Finally, while the study provides insights into behavioural determinants of food waste, it does not provide direct evidence for effective policy or educational interventions, which should therefore be interpreted cautiously.

For future research, expanding the dataset to include a more diverse and larger population sample would enhance generalizability. Incorporating real-time, sensor-based waste tracking and refining CNN algorithms to better differentiate food categories and volumes could improve accuracy. Furthermore, longitudinal studies could explore behaviour change over time and assess the long-term impact of interventions. Finally, integrating emotional or habitual dimensions into the TPB framework may yield deeper understanding of the psychological mechanisms driving food waste behaviour.

While the study offers valuable insights into the determinants of food waste behaviour among Generation Z, several limitations should be noted, particularly regarding generalizability. The sample primarily consisted of university students, which may not fully represent the broader young adult population across different educational, socio-economic, or geographic backgrounds. As such, findings should be interpreted cautiously when extrapolating to non-student or rural populations. Cultural and socio-economic factors, as well as differing familiarity with digital tools, may further influence diary and CNN-based data collection accuracy, potentially affecting cross-country comparability.

Despite these constraints, the integration of TPB-based surveys, food waste diaries, and CNN image analysis strengthens the reliability of behaviour assessment and highlights important behavioural patterns within this demographic. Future research should aim to include larger, more diverse samples of young adults, encompassing both university-attending and non-student populations across urban and rural settings, to enhance the generalizability of findings. Additionally, longitudinal and sensor-based studies could further validate behavioural trends and inform targeted policy or educational interventions.

The CNN model is primarily designed to recognise edible food items and does not reliably distinguish non-edible components such as peels, bones, shells, or other inedible fractions. As a result, waste quantities estimated from image analysis may include both edible and unavoidable food waste. To address this limitation, CNN-based estimates were interpreted at an aggregate category level and complemented by self-reported diary information, rather than being used as precise measures of avoidable food waste.

The use of a university-based convenience sample limits the generalizability of the results to the broader Generation Z population. Young adults not enrolled in higher education, as well as individuals from rural areas or different socio-economic backgrounds, may exhibit different food waste behaviours and motivations. Consequently, the findings should be interpreted as indicative of patterns within a higher-education subpopulation of Generation Z across different European contexts rather than as nationally representative estimates.

## 5. Conclusions

This study provides a comprehensive analysis of food waste behaviour among Generation Z participants in five European countries: Italy, Estonia, Croatia, Romania, and Serbia. Using an integrated multi-method approach—including food waste diaries, image recognition techniques, and survey data—we examined both actual food waste behaviours and the psychological factors driving the intention to avoid food waste.

The findings indicate significant cross-country differences in food waste quantities and types, with young Romanians discarding the highest proportion of food and Estonians the lowest. Meat and meat products (1.72%), ready meals (1.58%), and fruit (1.53%) were the most frequently wasted categories, though the specific types of food wasted varied by country.

The extended Theory of Planned Behaviour (TPB) model identified several key factors influencing young people’s intention to avoid food waste, including attitudes, subjective norms, perceived behavioural control, moral and social values, awareness of health risks, and the identity of being a good provider. Among these, intention, subjective norms, and provider identity were found to significantly influence actual food waste behaviour.

These results suggest that interventions should target both intention formation and behavioural execution. Strategies that strengthen perceived behavioural control, reshape social norms, and reinforce sustainable self-identity may be effective in reducing food waste. The educational programmes and public policies should be tailored to address both the most commonly discarded food categories and the regional variations in food waste patterns to foster more sustainable consumption habits among young people across Europe.

## Figures and Tables

**Table 1:** Participants’ demographic parameters (N = 330).

Parameters	Classes	Percent
Gender	Female	60.8
	Male	39.2
Living Arrangement	Alone	15.6
	With family	44.9
	With Roommate	37.4
	Other	2.1

**Table 2:** Average 7-day food waste measured by participant’s diary, expressed by portion of FW, and food waste quantity evaluated using the CNN model from 7-day food waste measured by participant’s image collection, expressed in percentage of FW.

Category	Italy		Estonia		Croatia		Romania		Serbia	
Number of Images/Plates	2780		2634		2832		2816		2722	
	Portion of FW	Percentage of FW	Portion of FW	Percentage of FW	Portion of FW	Percentage of FW	Portion of FW	Percentage of FW	Portion of FW	Percentage of FW
Fruit	0.54	2.07	0.25	0.50	1.25	2.37	0.55	1.12	0.89	1.59
Vegetables	0.26	0.70	0.34	0.60	0.63	1.94	0.38	1.16	0.93	2.94
Processed fruits and vegetables	0.12	0.34	0.06	0.16	0.37	0.81	0.08	0.14	0.28	0.78
Potatoes	0.17	0.76	0.04	0.15	0.35	0.54	0.60	2.75	0.37	0.51
Pasta, rice, cereal	0.26	0.92	0.06	0.23	0.42	0.74	0.34	1.24	0.34	1.21
Meat and meat products	0.19	0.81	0.23	0.60	0.79	3.15	1.06	2.18	0.66	1.84
Fish	1.28	4.52	0.01	0.01	0.20	0.85	0.05	0.22	0.11	0.32
Milk and dairy products	0.40	1.24	0.01	0.03	0.16	0.33	0.16	0.74	0.19	0.51
Bread	0.10	0.17	0.04	0.13	0.16	0.45	0.24	0.39	0.33	0.51
Cookies	0.06	0.23	0.00	0.00	0.06	0.16	0.17	0.71	0.04	0.09
Prepared meals	0.08	0.30	0.06	0.18	0.36	1.41	1.15	4.64	0.53	1.35
Other	0.20	0.47	0.30	0.84	0.51	1.78	0.61	1.89	0.49	0.92
Total	3.66	12.53	1.40	3.43	5.26	14.53	5.39	17.18	5.16	12.57

**Table 3:** The score of differences between diary and images by countries and correlation between food waste results obtained by participant’s diaries and image collections.

Methodology	Diary		Images		Diaries/Images
Category	Best	Worst	Best	Worst	Correlation
(1) Fruit	EST	CRO	EST	CRO	0.800
(2) Vegetables	EST	SRB	EST	SRB	0.987
(3) Processed fruits and vegetables	**EST**	CRO	**ROM**	CRO	0.968
(4) Potatoes	EST	ROM	EST	ROM	0.821
(5) Pasta, rice, cereal	EST	ROM	EST	ROM	0.708
(6) Meat and meat products	EST	**ROM**	EST	**CRO**	0.824
(7) Fish	EST	ITA	EST	ITA	0.999
(8) Milk and dairy products	EST	ITA	EST	ITA	0.941
(9) Bread	EST	SRB	EST	SRB	0.879
(10) Cookies	EST	ROM	EST	ROM	0.980
(11) Prepared meals	EST	ROM	EST	ROM	0.982
(12) Other	**ITA**	ROM	**EST**	ROM	0.883
Total	EST	ROM	EST	ROM	0.800

Abbreviations in bold show different results between diaries and pictures.

**Table 4:** Descriptive statistics and ANOVA table of constructs.

Construct	No. Items	Italy	Estonia	Croatia	Romania	Serbia	F	*p*
GWK (General waste knowledge)	19	2.718 ^a^	2.773 ^ab^	2.862 ^b^	2.728 ^a^	2.689 ^a^	6.087	0.000
GA (General attitude)	4	3.019 ^ab^	3.411 ^bc^	3.511 ^c^	2.960 ^a^	2.736 ^a^	11.453	0.000
MC (Moral criteria)	5	2.513 ^bc^	2.757 ^c^	2.588 ^c^	1.924 ^a^	2.175 ^ab^	11.861	0.000
I (Intention)	6	2.632 ^ab^	3.297 ^c^	2.830 ^b^	2.188 ^a^	2.212 ^a^	12.939	0.000
B (Behaviour)	5	2.521 ^abc^	2.424 ^bc^	2.111 ^a^	2.594 ^c^	2.153 ^ab^	4.352	0.001
PBC (Perceived behavioural control)	4	2.292 ^a^	2.429 ^a^	2.146 ^a^	2.183 ^a^	2.041 ^a^	1.869	0.099
SN (Subjective norm)	4	2.245 ^a^	2.244 ^a^	2.091 ^a^	1.881 ^a^	2.036 ^a^	1.858	0.101
FC (Financial concern)	3	3.403 ^b^	3.706 ^b^	3.621 ^b^	3.381 ^b^	2.739 ^a^	8.948	0.000
PHR (Perceived health risk)	5	2.616 ^bc^	2.897 ^c^	2.533 ^bc^	2.471 ^ab^	2.182 ^a^	5.946	0.000
PH (Planning habit)	3	2.736 ^bc^	3.103 ^c^	2.655 ^b^	2.609 ^ab^	2.316 ^a^	7.429	0.000
GPI (Good provider identity)	3	2.472 ^ab^	2.905 ^b^	2.531 ^ab^	2.1968 ^a^	2.231 ^a^	5.149	0.000

Different lower-case letters in the same row indicate significantly different values (*p* ˂ 0.05), according to post hoc Tukey’s HSD test.

**Table 5:** Cronbach’s alpha of constructs, average variance extracted and construct reliability.

Construct	No. of Items	Cronbach’s α	Average Variance Extracted	Construct Reliability
GA (General attitude)	4	0.844	0.590	0.850
MC (Moral criteria)	5	0.943	0.771	0.994
I (Intention)	6	0.939	0.727	0.941
B (Behaviour)	5	0.910	0.678	0.913
PBC (Perceived behavioural control)	4	0.913	0.734	0.917
SN (Subjective norm)	4	0.932	0.775	0.932
FC (Financial concern)	3	0.875	0.736	0.886
PHR (Perceived health risk)	5	0.865	0.683	0.915
GPI (Good provider identity)	3	0.808	0.612	0.824

**Table 6:** Correlation analysis between survey data, diaries and CNN image analysis of food waste.

		TPB							
		*GA*	*MC*	*I*	*B*	*PBC*	*SN*	*PHR*	*GPI*
Dairy data	Italy	0.534	0.669	0.754	0.482	0.690	0.678	0.696	0.682
	Estonia	0.521	0.662	0.768	0.471	0.668	0.668	0.697	0.683
	Croatia	0.520	0.681	0.753	0.496	0.666	0.657	0.680	0.682
	Romania	0.512	0.676	0.769	0.471	0.677	0.656	0.682	0.695
	Serbia	0.503	0.659	0.762	0.486	0.680	0.668	0.687	0.681
CNN image analysis	Italy	0.549	0.655	0.754	0.469	0.688	0.667	0.676	0.708
	Estonia	0.531	0.676	0.761	0.465	0.683	0.656	0.693	0.677
	Croatia	0.530	0.651	0.753	0.457	0.671	0.648	0.686	0.694
	Romania	0.495	0.660	0.767	0.470	0.704	0.650	0.682	0.701
	Serbia	0.518	0.641	0.769	0.482	0.693	0.651	0.684	0.691

## Data Availability

The data presented in this study are available on request from the corresponding author.

## Supplementary Materials

The following supporting information can be downloaded at: https://www.mdpi.com/article/10.3390/foods15040696/s1, Figure S1: Food waste measurements, Figure S2: Correspondence analysis presentation of average 7-day food waste measured by participant’s diary/image collection, expressed by portion, Table S1: Survey questions, Table S2: General waste knowledge and opinion, Table S3: General waste knowledge and opinion (%), Table S4: General waste knowledge and opinion, Table S5: General Attitude toward food waste, Table S6: EFA—Rotated component matrix, Table S7: Standardised regression weights of variables, Table S8: Discriminant Validity, Table S9: Correlation analysis between constructs, Table S10: Path coefficients, S. E. values, C and Results of the Model, Table S11: Constructs as mediators.

## Abbreviations

The following abbreviations are used in this manuscript:

FW	Food waste
TPB	Theory of Planned Behaviour
SEM	Structural Equation Modelling
AGFI	advanced goodness of fit
B	Food waste behaviour
CFI	Comparative Fit Index
FC	Financial concern
GFI	Goodness of fit
GPI	Good provider identity
GWK	General attitude regarding FW
I	Intention
MC	Moral criteria
PBC	Perceived behaviour control
PHR	Perceived health risk
SN	Subjective norm
RMSE	Root Mean Square Error of Approximation
NFI	Normed Fit Index
TLI	Tucker–Lewis Index
