# Supplementary material for: An Analysis of Food Waste Production and Behavioural Patterns Among Generation Z in Five European Countries
Source: Foods. 2026 Feb 13;15(4):696. doi: 10.3390/foods15040696 (PMC12939773; doi:10.3390/foods15040696)
Supplement: Supplementary file 1 [file foods-15-00696-s001.zip › foods-4134004-supplementary.pdf]

foods

Article

An Analysis of Food Waste Production and Behavioural Patterns Among Generation Z in Five European Countries

Neven Voća[1], Francesco Donsi[2,*], Mirela Alina Sandu[3], Viktoria Voronova[4], Jana Šic Žlabur[1], Giovanni De Feo[2], Ana Virsta[3], Marija Klõga[4], Jelena Lubura Stošić[5], Anamarija Peter[1], Gina Vasile Scăețeanu[6], Sanja Ostojić[7], Ivan Brandić[1], Gianpiero Pataro[2], Dario Balaban[5], Darko Micić[7], Jona Šurić[1], Saša Đurović[7,8], Alessandra Procentese[2] and Lato Pezo[7,*]

1 Faculty of Agriculture, University of Zagreb, Svetošimunska cesta 25, 10000 Zagreb, Croatia; nvoca@agr.hr (N.V.); jszlabur@agr.hr (J.Š.Ž.); apeter@agr.hr (A.P.); jsuric@agr.hr (J.Š.)

2 Department of Industrial Engineering, University of Salerno, via Giovanni Paolo II, 132, 84084 Fisciano, Italy; g.defeo@unisa.it (G.D.F.); gpataro@unisa.it (G.P.); aprocentese@unisa.it (A.P.)

3 Faculty of Land Reclamation and Environmental Engineering, University of Agronomic Sciences and Veterinary Medicine of Bucharest, 59 Marasti Blvd., District 1, 011464 Bucharest, Romania; mirela.sandu@fifim.ro (M.A.S.); ana.virsta@fifim.ro (A.V.)

4 Department of Civil Engineering and Architecture, Tallinn University of Technology, Ehitajate tee 5, 19086 Tallinn, Estonia; viktoria.voronova@taltech.ee (V.V.); marija.kloga@taltech.ee (M.K.)

5 Faculty of Technology Novi Sad, University of Novi Sad, Bul. Cara Lazara 1, 21000 Novi Sad, Serbia; jelenalubura@uns.ac.rs (J.L.S.); dariob961@gmail.com (D.B.)

6 Faculty of Agriculture, University of Agronomic Sciences and Veterinary Medicine of Bucharest, 59 Marasti Blvd., District 1, 011464 Bucharest, Romania; ginavasile2000@yahoo.com

7 Institute of General and Physical Chemistry, University of Belgrade, Studentski Trg 12-16, 11000 Beograd, Serbia; ostojicsanja404@gmail.com (S.O.); micic83@gmail.com (D.M.); sasatfns@uns.ac.rs (S.Đ.)

8 Institute of Biomedical Systems and Biotechnology, Peter the Great St-Petersburg Polytechnic University, Khlopina d. 11, korp. 1, Lit. A, 195251 Saint-Petersburg, Russia

* Correspondence: fdonsi@unisa.it (F.D.); latopezo@yahoo.co.uk (L.P.)

Supplement materials

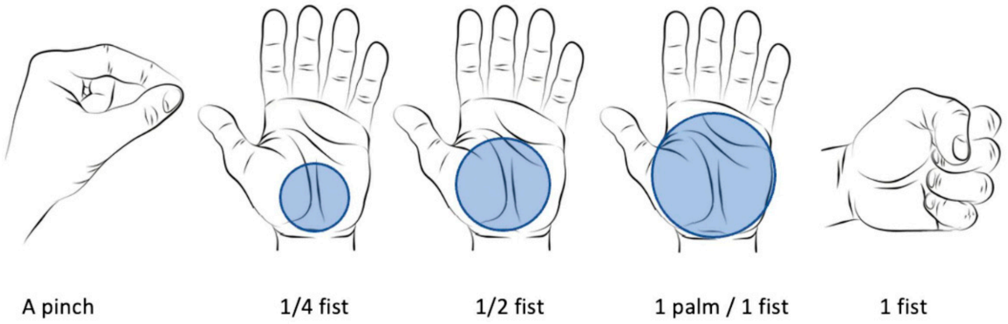

Supplement Figure S1. Food waste measurements

Supplement Table S1. Survey questions

	GEN	Gender:
	CNT	Country:
	FAC	The name of the faculty you are attending:
	LIV	Living Arrangement:
**GWK**	GWK1	During your education have you ever been educated about proper waste management?
	GWK2	Do you think that waste if not disposed of properly can pollute the environment?
	GWK3	In your estimation what is the average daily amount of municipal waste generated per capita in EU?
	GWK4	Depending on the characteristics the waste can be hazardous non-hazardous or inert.
	GWK5	The abbreviation RDF stands for?
	GWK6	Which waste management principles according to waste management hierarchy is considered the least desirable?
	GWK7	Biowaste represents a great potential for gas production.
	GWK8	How much food produced worldwide is lost or wasted every year.
	GWK9	Compost is a useful addition to the soil.
	GWK10	What waste material cannot be composted?
	GWK11	Vegetarian diets produce less food waste than diets with meat.
	GWK12	What do you think about the incineration of waste?
	GWK13	Separate waste collection is necessary in every participant cafeteria.
	GWK14	I understand the difference between the best before date and the use by date on food items.
	GWK15	A best before date means that food from that date onwards can become a health risk and should therefore no longer be consumed.
	GWK16	Do you think participants should be educated more about how to deal with food waste?
**GA**	GA1	Food waste is unavoidable - I produce food waste every day.
	GA2	It disgusts me that unused produce ends up in the trash bin.
	GA3	It is better to waste food than to gain weight by eating too much.
	GA4	It is better to waste food than to risk infection by eating produce that is not fresh.
**MC**	MC1	It is immoral to waste food while some people are starving.
	MC2	I feel bad when I waste food.
	MC3	I feel obliged not to waste food.
	MC4	Wasting food is against my principles.
	MC5	My religion forbids me to waste food.
**I**	I1	I intend to avoid wasting food as much as possible.
	I2	I plan to eat all the food that I buy or prepare,
	I3	I intend to reuse my leftovers when preparing my next meals,
	I4	I intend to make use of my friends' or colleagues' leftovers when possible,
	I5	I intend to eat leftovers from my friends' or colleagues' plate if appropriate,
	I6	I do not intend to throw away food that is still edible, even if it does not taste perfect.
**B**	B1	I usually order more food than I can eat in the cafeteria,
	B2	I often buy food that I do not end up consuming,
	B3	Before ordering, I consider the price of food to decide how much I take and whether I will finish it,
	B4	I plan my meals in advance and usually follow that plan to avoid wasting food,
	B5	I often buy cheaper food so that throwing it away concerns me less.
**PBC**	PBC1	For me, reducing food waste is easy to do,
	PBC2	I feel confident in my ability to avoid wasting food,
	PBC3	Whether I waste food or not is completely up to me,
	PBC4	I have the resources and opportunities needed to reduce food waste.
**SN**	SN1	People who are important to me think I should reduce food waste,
	SN2	Most people like me try to avoid wasting food,
	SN3	I feel social pressure from friends or family to reduce food waste,
	SN4	People whose opinions I value would approve of me reducing food waste.
**FC**	FC1	I think wasting food is a waste of money.
	FC2	Saving money motivates me to throw away less food.
	FC3	I rarely think of money when I throw away food in the participant cafeteria.
**PHR**	PHR1	Would you consume a product from the participant canteen at a lower price that is about to expire?
	PHR2	I am not worried that eating leftovers could lead to poor health
	PHR3	I believe that the risk of illness from eating food after the expiry date is high

PH	PH1	I prepare for my food order by exploring the online menu of my participant cafeteria or restaurants
	PH2	I consider myself a person who loves to plan things
	PH3	I plan to buy exactly as much food as I can eat.
**GPI**	GPI1	When I prepare a party or dinner for my friends, I feel uncomfortable if my guests eat up all the food I have prepared. They probably want to eat more
	GPI2	When I prepare a party or dinner for my friends, I like to offer a wide variety of food so that everyone has something he or she likes
	GPI3	When I prepare a party or dinner for my friends, I like to buy more food than necessary because I am a generous host

Supplement Table S2. General waste knowledge and opinion

Answer	Question (%)								
	GWK1	GWK2	GWK4	GWK7	GWK9	GWK11	GWK13	GWK14	GWK15
I don't know	9.281	2.994	16.168	8.683	5.090	20.958	2.695	10.479	8.982
No	29.341	1.796	2.695	3.293	2.096	33.234	3.593	7.186	47.904
Yes	61.377	95.210	81.138	88.024	92.814	45.808	93.713	82.335	43.114

Supplement Table S3. General waste knowledge and opinion (%)

**GWK3**	**Answer**	**GW5**	**Answer**	**GWK6**	**Answer**	**GWK8**	**Answer**	**GWK10**	**Answer**
11.4	2-3 kg	31.4	I don't know	21.0	Prevention	15.3	1:2	76.9	Bones
32.3	1-2 kg	28.4	Recycling domestic factory	40.7	Recycling	51.2	1:3	11.7	Egg shells
32.3	0.5-0.7 kg	32.6	Refuse derived fuel	18.3	Reuse	20.7	2:3	2.1	Vegetables
11.7	0.15-0.25 kg	7.5	Reuse domestic facility	20.1	I don't know	12.9	I don't know	9.3	I don't know
12.3	I don't know	31.4	I don't know						

Supplement Table S4. General waste knowledge and opinion

**GWK12 (%)**	**Answer**	**GWK16 (%)**	**Answer**
35.0	Positive	92.5	Yes
49.4	Negative	1.8	There is no need - they already know enough
15.6	I don't know	0.9	No
		4.8	I don't know

Supplement Table S5. General Attitude toward food waste

Answer	GA1 (%)	GA2 (%)	GA3 (%)	GA4 (%)
1	11.078	4.491	34.731	8.982
2	21.557	10.180	23.952	28.743
3	21.257	23.353	27.246	19.461
4	36.527	32.934	9.880	25.749
5	9.581	29.042	4.192	17.066

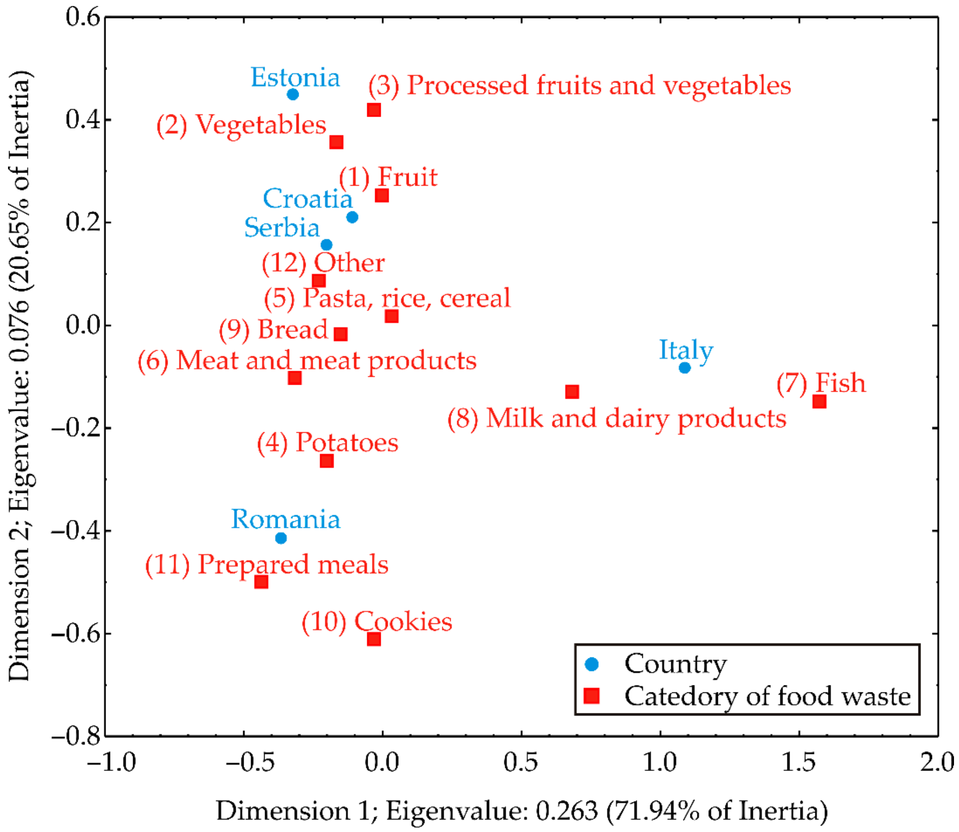

Supplement Figure S2. Correspondence analysis presentation of average 7-day food waste measured by participant's diary / image collection, expressed by portion

Supplement Table S6. EFA - Rotated component matrix

	Component									
	1	2	3	4	5	6	7	8	9	10
GA1							0.752			
GA2							0.811			
GA3							0.796			
GA4							0.728			
MC1				0.768						
MC2				0.684						
MC3				0.749						
MC4				0.729						
MC5				0.881						
I1		0.757								
I2		0.794								
I3		0.829								
I4		0.853								
I5		0.794								
I6		0.628								
B1			0.804							
B2			0.822							
B3			0.739							
B4			0.810							
B5			0.932							
PBC1					0.718					

PBC2					0.858					
PBC3					0.801					
PBC4					0.768					
SN1						0.848				
SN2						0.781				
SN3						0.733				
SN4						0.650				
FC1									0.872	
FC2									0.851	
FC3									0.780	
PHR1		0.622								
PHR2		0.566						0.549		
PHR3								0.845		
~~PH1~~							~~0.703~~	~~0.703~~		
~~PH2~~							~~0.713~~			
~~PH3~~	~~0.782~~									
GPI1										0.688
GPI2										0.646
GPI3										0.694

GA - General attitude, MC - Moral criteria, I – Intention, B – Behaviour, PBC - Perceved behavioural control, SN - Subjective norm, FC - Financial concern, PHR - Perceived health risk, GPI (Good provider identity)

Supplement Table S7. Standardized regression weights of variables

Question	GA	FC	MC	PBC	PHR	SN	I	GPI	B
1	0.715	0.979	0.827	0.807	0.795	0.952	0.847	0.93	0.782
2	0.944	0.868	0.897	0.866	0.813	0.901	0.827	0.876	0.79
3	0.651	0.682	0.908	0.885	0.853	0.855	0.864	0.893	0.74
4	0.732		0.917	0.867		0.808	0.961		0.818
5			0.837				0.885		0.969
6							0.731		

GA - General attitude, MC - Moral criteria, I – Intention, B – Behaviour, PBC - Perceved behavioural control, SN - Subjective norm, FC - Financial concern, PHR - Perceived health risk, GPI (Good provider identity)

Supplement Table S8. Discriminant Validity

			Correlation	Squared correlation	AVE1	AVE2
FC	<-->	GWK	0.13	0.017	0.736	0.560
MC	<-->	GWK	-0.025	0.001	0.771	0.560
PBC	<-->	GWK	-0.057	0.003	0.734	0.560
PHR	<-->	GWK	-0.024	0.001	0.683	0.560
SN	<-->	GWK	-0.098	0.010	0.775	0.560
GA	<-->	GWK	0.116	0.013	0.590	0.560
I	<-->	GWK	-0.029	0.001	0.729	0.560
B	<-->	GWK	-0.081	0.007	0.678	0.560
GPI	<-->	GWK	-0.074	0.005	0.810	0.560
GA	<-->	FC	0.466	0.217	0.590	0.736
GA	<-->	MC	0.429	0.184	0.590	0.771
GA	<-->	PBC	0.235	0.055	0.590	0.734

GA	<-->	PHR	0.377	0.142	0.590	0.683
GA	<-->	SN	0.141	0.020	0.590	0.775
GA	<-->	I	0.422	0.178	0.590	0.729
GA	<-->	B	0.037	0.001	0.590	0.678
GA	<-->	GPI	0.271	0.073	0.590	0.810
FC	<-->	MC	0.313	0.098	0.736	0.771
FC	<-->	PBC	0.255	0.065	0.736	0.734
FC	<-->	PHR	0.348	0.121	0.736	0.683
FC	<-->	SN	0.138	0.019	0.736	0.775
I	<-->	FC	0.314	0.099	0.729	0.736
GPI	<-->	FC	0.265	0.070	0.810	0.736
B	<-->	FC	0.152	0.023	0.678	0.736
MC	<-->	PBC	0.472	0.223	0.771	0.734
MC	<-->	PHR	0.595	0.354	0.771	0.683
MC	<-->	SN	0.546	0.298	0.771	0.775
I	<-->	MC	0.73	0.533	0.729	0.771
B	<-->	MC	0.207	0.043	0.678	0.771
GPI	<-->	MC	0.541	0.293	0.810	0.771
PBC	<-->	PHR	0.455	0.207	0.734	0.683
PBC	<-->	SN	0.514	0.264	0.734	0.775
I	<-->	PBC	0.475	0.226	0.729	0.734
B	<-->	PBC	0.438	0.192	0.678	0.734
GPI	<-->	PBC	0.7	0.490	0.810	0.734
PHR	<-->	SN	0.59	0.348	0.683	0.775
I	<-->	PHR	0.775	0.601	0.729	0.683
B	<-->	PHR	0.336	0.113	0.678	0.683
GPI	<-->	PHR	0.494	0.244	0.810	0.683
I	<-->	SN	0.637	0.406	0.729	0.775
B	<-->	SN	0.371	0.138	0.678	0.775
GPI	<-->	SN	0.507	0.257	0.810	0.775
B	<-->	I	0.26	0.068	0.678	0.729
I	<-->	GPI	0.629	0.396	0.729	0.810
B	<-->	GPI	0.244	0.060	0.678	0.810

GA - General attitude, MC - Moral criteria, I – Intention, B – Behaviour, PBC - Perceved behavioural control, SN - Subjective norm, FC - Financial concern, PHR - Perceived health risk, GPI (Good provider identity)

Supplement Table S9. Correlation analysis between constructs

	MC	I	B	PBC	SN	FC	PHR	GPI
GA	0.114*	0,105	0,001	-0,040	0,084	0,056	0,060	0.108*
MC		0.663**	0.180**	0.398**	0.510**	0.273**	0.499**	0.480**
I			0.251**	0.455**	0.624**	0.326**	0.703**	0.577**
B				0.401**	0.352**	0.179**	0.285**	0.216**
PBC					0.446**	0.291**	0.370**	0.624**
SN						0.216**	0.568**	0.489**
FC							0.319**	0.305**
PHR								0.441**

* Correlation is significant at the 0.05 level (2-tailed), **. Correlation is significant at the 0.01 level (2-tailed).

GA - General attitude, MC - Moral criteria, I – Intention, B – Behaviour, PBC - Perceved behavioural control, SN - Subjective norm, FC - Financial concern, PHR - Perceived health risk, GPI (Good provider identity)

Supplement Table S10. Path coefficients, S. E. values, C and Results of the Model

Hypothesis	Path			Estimate	Standard error (S.E.).	Critical ratio (C.R)	p - value	Result
H1	I	<---	GA	0.098	0.042	2.342	0.019	Confirmed
H2	I	<---	MC	0.458	0.079	5.83	***	Confirmed
H3	I	<---	GPI	0.357	0.07	5.112	***	Confirmed
H4	I	<---	SN	0.247	0.068	3.643	***	Confirmed
H5	I	<---	PHR	0.733	0.088	8.311	***	Confirmed
H6	I	<---	PBC	-0.209	0.081	-2.592	0.01	Confirmed
H7	B	<---	I	0.146	0.062	2.358	0.018	Confirmed
H8	B	<---	GPI	0.175	0.086	2.036	0.042	Confirmed
H9	GPI	<---	MC	0.273	0.064	4.25	***	Confirmed
H10	GPI	<---	PBC	0.64	0.067	9.595	***	Confirmed
H11	GPI	<---	SN	0.118	0.059	1.989	0.047	Confirmed

*** $p<0.001$

GA - General attitude, MC - Moral criteria, I – Intention, B – Behaviour, PBC - Perceved behavioural control, SN - Subjective norm, FC - Financial concern, PHR - Perceived health risk, GPI (Good provider identity)

Supplement Table S11. Constructs as mediators

			a	SE_a	b	SE_b	a × b	SE_{ab}	z value	Result
MC	GPI	B	0.273	0.064	0.175	0.086	0.048	0.029	1.663	
PBC	GPI	B	0.64	0.067	0.175	0.086	0.112	0.030	3.772	Confirmed
SN	GPI	B	0.118	0.059	0.175	0.086	0.021	0.027	0.761	
MC	I	B	0.458	0.079	0.146	0.062	0.067	0.026	2.544	Confirmed
GWK_2	I	B	0.098	0.042	0.146	0.062	0.014	0.016	0.869	
GPI	I	B	0.357	0.07	0.146	0.062	0.052	0.024	2.178	Confirmed
SN	I	B	0.247	0.068	0.146	0.062	0.036	0.023	1.540	
PHR	I	B	0.733	0.088	0.146	0.062	0.107	0.029	3.741	Confirmed
PBC	I	B	-0.209	0.081	0.146	0.062	-0.031	0.027	-1.139	
